# Assessing nutritional risk and its association with mortality in ICU patients using the modified NUTRIC score: evidence from a tertiary care hospital

**DOI:** 10.1080/07853890.2026.2690708

**Published:** 2026-06-18

**Authors:** Burhan Ahmad, Iqra Akhtar, Muhammad Taimur, Syed Hassan Bin Usman Shah, Abdul Momin Rizwan Ahmad, Muhammad Irfan, Rohina Ghafori

**Affiliations:** a Department of Nutrition, Fauji Foundation Hospital, Rawalpindi, Pakistan; b Department of Nutrition & Dietetics, National University of Medical Sciences (NUMS), Rawalpindi, Pakistan; c Department of Surgery, Fauji Foundation Hospital, Rawalpindi, Pakistan; d Department of Public Health, PRIME Consulting, Islamabad, Pakistan; e Department of Human Nutrition and Dietetics, NUST School of Health Sciences, National University of Sciences & Technology (NUST), Islamabad, Pakistan; f Department of Health Sciences, University of York, York, United Kingdom; g Department of Statistics, Liaquat National Hospital and Medical College, Karachi, Pakistan; h United Nations Development Programme, Afghanistan

**Keywords:** Critically ill, intensive care unit, modified NUTRIC, malnutrition, mortality, nutritional risk

## Abstract

**Background:**

The objective of this study was to assess nutritional risk in ICU patients using the modified Nutritional Risk in Critically ill (mNUTRIC) score and to evaluate its association with in-hospital mortality.

**Methods:**

A cross-sectional study was conducted in a tertiary care hospital, including 584 ICU admitted patients. The mNUTRIC score was calculated, and the association between mNUTRIC-defined nutritional risk and mortality was evaluated using logistic regressions.

**Results:**

Of the total 584 patients, the mean 57.6 ± 15.3 years and 67.5% (*n* = 394) were female. The mean APACHE-II and SOFA scores were 18.9 ± 8.5 and 5.1 ± 1.9, respectively. The mean mNUTRIC score was 3.2 ± 2.0 with 69.9% of patients classified as low nutritional risk and 30.1% as high nutritional risk. Patients classified as having high nutritional risk by the mNUTRIC score had a high mortality rate (62.9%). In adjusted mortality model, low nutritional risk based on the mNUTRIC score was associated with significantly lower odds of mortality (aOR 0.51, 95% CI 0.28–0.91) after adjustment for APACHE-II score, the number of comorbidities, and ICU type. The area under the curve for discriminating mortality was 0.819.

**Conclusion:**

The mNUTRIC score is a useful nutritional risk screening tool and demonstrates a strong prognostic association with mortality in critically ill ICU patients, supporting its applicability in a tertiary care hospital context in Pakistan.

## Introduction

Malnourished patients frequently experience adverse clinical outcomes, including increased morbidity and mortality, posing a significant challenge for intensive care unit (ICU) healthcare practitioners [1,2]. ICU admission is often associated with a hypermetabolic state, coupled with factors such as a pronounced inflammatory response, delayed initiation of nutritional support, and interruptions in nutrient delivery, accelerating malnutrition in critically ill patients [3,4]. Malnutrition impacts not only cellular function and body composition but also key outcomes like wound healing, infection rates, difficulties in weaning from mechanical ventilation, ICU length of stay, and increased healthcare costs. Furthermore, the mortality rates among ICU patients are markedly elevated [3]. Consequently, early and accurate nutritional risk assessment becomes crucial in managing critically ill patients.

Assessing nutrition requirements is a cornerstone for eliminating the risk of malnutrition [3]. Given that malnutrition is potentially reversible, accurate and timely nutritional risk screening is crucial in ICU care. Early nutritional support helps reduce hospital malnutrition and mitigates adverse outcomes, including oxidative cellular damage [1]. Despite extensive research emphasizing the importance of adequate nutritional support for critically ill patients, nutritional delivery remains suboptimal, and the prevalence of hospital malnutrition has remained largely unchanged. The prevalence of malnutrition in critically ill patients varies between 37% and 78%, depending on the patient population and the screening methods [5,6]. In Pakistan, 45% of mechanically ventilated ICU patients are at high nutritional risk, which correlates with longer ICU stays and higher mortality [7].

The absence of standardized nutrition guidelines is a key barrier to effective nutrition assessment. The 2019 European Society for Clinical Nutrition and Metabolism (ESPEN) guidelines [8] recommended that all ICU patients admitted for more than 48 h undergo nutritional evaluation. Implementing a systematic, evidence-based approach to nutrition assessment is crucial for optimizing outcomes in critically ill patients. The mNUTRIC score (modified Nutrition Risk in Critically Ill) is a nutritional risk screening tool designed to identify critically ill patients who may benefit most from nutritional therapy [9–11]. Undeniably, patients with a high mNUTRIC score are at increased risk of adverse outcomes, longer hospital stays, and higher mortality, and are more likely to benefit from intensive nutritional intervention [6,9].

Although the mNUTRIC score has been widely validated in various ICU settings globally, evidence on the prognostic performance and practical applicability in low-resource settings like Pakistan remains limited, highlighting the need for context-specific data to guide the nutritional risk assessment and early intervention strategies. The main objective of our study was to assess whether the modified NUTRIC score is an effective tool for evaluating nutritional risk and its association with mortality among patients admitted to the ICU of a tertiary care hospital in Rawalpindi, Pakistan.

## Methods

### Study design and population

A prospective observational cross-sectional study was performed from 1st January 2024 to 30th November 2024 in the ICU settings of one semi-governmental tertiary care hospital of Rawalpindi, Pakistan. Patients were recruited from both the surgical ICU (SICU) and the hospital’s Medical ICU (MICU). During this time period, *n* = 660 patients admitted to the ICUs were screened, of whom *n* = 584 met the eligibility criteria and were included in the analysis. The remaining 76 patients were excluded because they met one or more exclusion criteria: death within 24 h of ICU admission, discharge on request or leaving against medical advice, or transfer to another hospital before outcome assessment.

### Inclusion and exclusion criteria

The inclusion criteria were adult patients (aged ≥ 18 years), admitted to either SICU or MICU, and who stayed in the ICU for at least 24 h. Those patients who expired within 24 h after ICU admission were either given discharge on request or left against medical advice (LAMA); or patients transferred to another hospital were excluded from the study.

### Nutrition screening and data collection

Nutritional risk screening in critically ill patients was conducted by two trained nutritionists. However, only the senior nutritionist was involved in the final scoring of mNUTRIC score. The junior nutritionist was involved only with data collection. To identify the patients at nutritional risk, the mNUTRIC score (modified NUTRIC) was used. This validated tool quantifies the risk of critically ill patients developing adverse outcomes that may be improved by timely nutritional intervention [12]. The tool constitutes five variables, including age, number of co-morbidities, days from hospital admission to ICU transfer, Acute Physiology and Chronic Health Evaluation II (APACHE II) and Sequential Organ Failure Assessment (SOFA). ICU stay duration was categorized into three groups: 1–3 days, 4–7 days and >7 days. Since our inclusion criteria required a minimum ICU stay of 24 h, no patients with ICU stays <24 h were included. APACHE II records items to measure physiological variables, age points and chronic health points. SOFA measures different body systems, including the respiratory, coagulation, liver, cardiovascular, central nervous and renal systems. A total score of 5–9 was considered high risk for malnutrition, whereas a score between 0 and 4 was considered low risk [13]. The mNUTRIC score was calculated at the time of ICU admission using patients’ baseline clinical data before any outcome was known. In addition to the variables required to calculate the mNUTRIC score, data were collected on sex, ICU type, height, adjusted body weight, ideal body weight, ICU length of stay, mortality status and comorbidities. Height, adjusted body weight and ideal body weight were calculated as per the standard protocols for ICU patients. Body mass index was also calculated using the standard formula [14].

### Data analysis

Data analysis was performed using IBM SPSS Statistics v27 (IBM SPSS Statistics, Armonk, NY). Data normality was checked using the Shapiro-Wilk test. Continuous variables were summarized using mean ± standard deviation or median and inter-quartile range, as appropriate, and categorical variables were summarized using frequencies and percentages. Chi-square/Fisher’s exact test was applied to determine association between qualitative variables. Independent-samples *t*-tests were used to compare normally distributed continuous variables between nutritional risk groups and survival status groups, while Mann–Whitney U test was used for non-normally distributed variables. Univariate logistic regressions were used to assess relationships between patient characteristics and mortality, and results are reported as odds ratios (ORs) with 95% confidence intervals (CIs). For the mortality analysis, mNUTRIC-defined nutritional risk was specified as the primary exposure of interest. The adjusted mortality model included APACHE-II score, number of comorbidities, and ICU type as pre-specified covariates based on clinical relevance and their potential role as confounders of the nutritional risk–mortality relationship. The adjusted results are therefore presented for the mNUTRIC exposure only, while the adjustment variables are listed in the table footnote. For nutritional risk as the outcome, only unadjusted logistic regression analyses are reported because several variables associated with nutritional risk are also component variables of the mNUTRIC score and an adjusted model would not yield clearly interpretable estimates. Variance inflation factors (VIF) were examined to assess multicollinearity among covariates included in the adjusted mortality model. The discriminatory ability of the mNUTRIC score for the mortality was evaluated using receiver operating characteristic (ROC) curve analysis, and the Youden index was used to determine the optimal cut-off for high nutritional risk. *P* value less than 0.05 was considered as significant [15].

## Results

### Study participants

A total of 660 people admitted to the ICU were screened, and 584 people who met the inclusion criteria were enrolled in the study with a mean age of 57.6 ± 15.3 years. There were 67.5% (*n* = 394) female patients and 32.5% (*n* = 190) male patients. Among all the admitted patients, around 36.9% were under 50 years, 43.8% between 50 and 74 years and 19.2% above 74 years. The mean ICU stay was 4.3 ± 4.0 days. The mean body mass index was 21.5 ± 1.9 kg/m^2^. Majority of the patients 66.4% (*n* = 388) were admitted to the medical ICU. Hypertension (45.5%), ischemic heart disease (44.8%) and diabetes mellitus (35.1%) were the common co-morbidities among the admitted patients. The mean APACHE-II and SOFA scores were 18.9 ± 8.5 and 5.1 ± 1.9, respectively. The mean mNUTRIC score was 3.2 ± 2.0, with 69.9% of patients classified as low nutritional risk and 30.1% as high nutritional risk. The mortality rate among the admitted patients was 29.1% (Table 1).

**Table 1.** Demographic characteristics of ICU-admitted patients (*n* = 584).

Characteristics	*n* (%)
**Gender**	
Male	190 (32.5)
Female	394 (67.5)
**Age (years)**	
Mean ± SD	57.6 ± 15.28
**Age groups**	
<50 years	216 (36.9)
50–74 years	256 (43.8)
>74 years	112 (19.2)
**Height (cm)**	165 ± 7.95
**Ideal Body Weight (kg), *n* = 563**	58.81 ± 9.44
**Adjusted Body Weight (kg), *n* = 21**	65.28 ± 20.95
**Body Mass Index (kg/m^2^)**	21.50 ± 1.99
**ICU stay (days)**	4.39 ± 4.09
**Groups (ICU stay days)**	
≤3 days	327 (56)
4–7 days	169 (28.9)
>7 days	88 (15.1)
**Length of hospital stay to ICU transfer (days)**	
Mean ± SD	1.39 ± 2.18
**Groups (hospital stay to ICU transfer)**	
0 days	380(65.1)
≥1 days	204(34.9)
**APACHE-II score**	
Mean ± SD	18.91 ± 8.51
**APACHE-II sub-groups**	
<15	197 (33.7)
15–19	132 (22.6)
20–28	138 (23.6)
≥28	117 (20)
**SOFA score**	
Mean ± SD	5.12 ± 1.98
**SOFA sub-groups**	
<6	414 (70.9)
6–9	157 (26.9)
≥10	13 (2.2)
**No. of co-morbidities**	
Mean ± SD	1.47 ± 1.44
**Co-morbidities** sub-groups	
≤1	303 (51.9)
>1	281 (48.1)
**Co-morbidities**	
Ischemic heart disease	262 (44.8)
Hypertension	266 (45.5)
Diabetes Mellitus	205 (35.1)
Chronic kidney disease	77 (13.2)
Asthma	23 (3.9)
Sepsis	26 (4.4)
**ICU**	
Medical ICU	388 (66.4)
Surgical ICU	196 (33.6)
**Mortality**	
No	414 (70.9)
Yes	170 (29.1)
**mNUTRIC Score**	
Mean ± SD	3.26 ± 2.07
**Nutritional risk by mNUTRIC score**	
Low nutritional risk (between 0 and 5)	408 (69.9)
High nutritional risk (>5)	176 (30.1)

**Figure 1.**
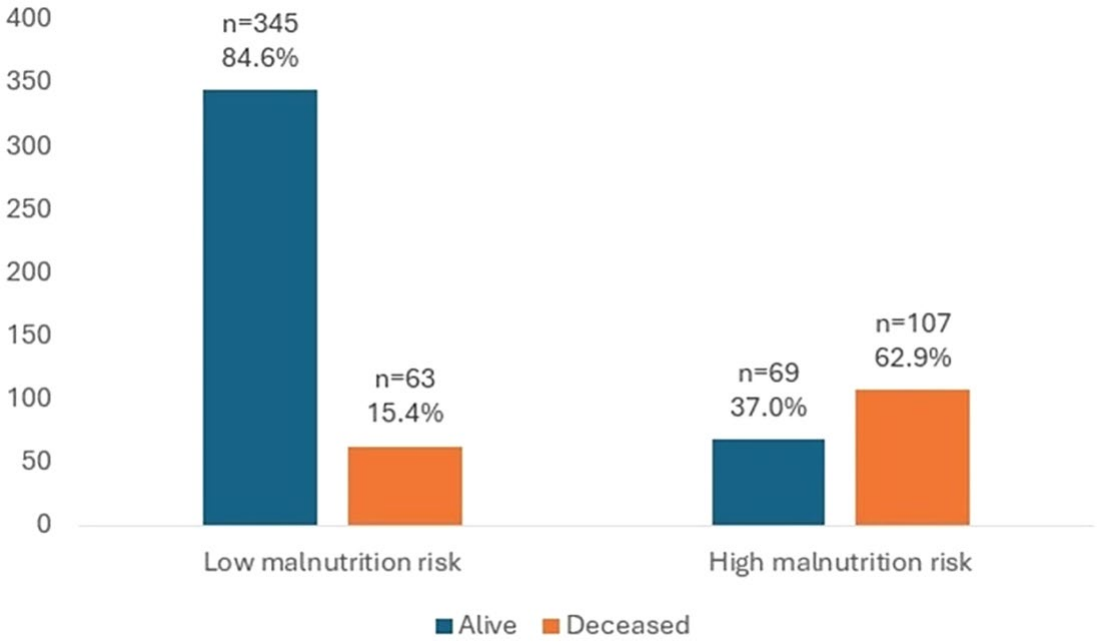
Mortality according to the nutritional risk by mNUTRIC score.

Figure 1 shows a high mortality rate (62.9%) among patients with a high nutritional risk as identified by the mNUTRIC score, noting that this association likely reflects, in part, the contribution of illness severity components (APACHE-II and SOFA) embedded within the score.

### Nutritional risk, severity scores and mortality outcomes in ICU patients

The median age of patients at low nutritional risk was 50.00 (IQR = 25.00) years compared to 70.00 (IQR = 21.00) years of patients at high risk. Compared to the patients with low nutritional risk, those at high nutritional risk exhibited higher median SOFA and APACHE-II scores (7.00 vs. 4.00 and 29.00 vs. 15.00, respectively) and demonstrated a higher mNUTRIC score (6.00 vs. 2.00).

Patients who died during their ICU admission had higher median SOFA scores (6.00 vs. 4.00), APACHE II scores (29.00 vs. 16.00), and mNUTRIC scores (5.00 vs. 3.00) than those who survived (Table 2).

**Table 2.** Mean comparison of clinical characteristics based on mNUTRIC score and survival status.

	Nutritional risk by mNUTRIC score			Mortality		
Characteristics	Low Risk	High Risk	*p* value	No	Yes	*p* value
Age (years)	53.6 ± 14.33	66.85 ± 13.28	<0.001*	56 ± 15.3	61.48 ± 14.56	<0.001*
ICU stay (days)	4.33 ± 4.01	4.53 ± 4.27	0.583	4.2 ± 4.03	4.85 ± 4.2	0.078
Length of hospital stay to ICU transfer (days)	1.24 ± 2.08	1.75 ± 2.35	0.014*	1.36 ± 2.18	1.47 ± 2.18	0.603
APACHE-II score	15.24 ± 7.02	27.43 ± 4.71	<0.001*	16.06 ± 7.42	25.88 ± 6.81	<0.001*
SOFA Score	4.45 ± 1.45	6.67 ± 2.16	<0.001*	4.64 ± 1.64	6.3 ± 2.22	<0.001*
Number of co-morbidities	1.13 ± 1.32	2.26 ± 1.39	<0.001*	1.35 ± 1.4	1.77 ± 1.49	0.002*
Body Mass Index	21.45 ± 1.95	21.63 ± 2.08	0.315	21.53 ± 2.1	21.43 ± 1.68	0.571
mNUTRIC Score	2.2 ± 1.45	5.73 ± 0.82	<0.001*	2.6 ± 1.91	4.88 ± 1.48	<0.001*

Independent *t* test was applied.

*p* value less than 0.05 were considered as significant.

* Significant at 0.05 level.

### Factors associated with nutritional risk and mortality

The factors associated with nutritional risk and mortality are presented in Table S1.

Unadjusted regression analysis for high nutritional risk, as determined by the mNUTRIC score, is presented in Table 3. Unadjusted mortality analyses and the adjusted association between mNUTRIC-defined nutritional risk and mortality are presented in Table 4.

**Table 3.** Unadjusted analysis of factors associated with high nutritional risk by mNUTRIC score following ICU admission (*n* = 584).

		Unadjusted	
Variable/category	N	OR (95% CI)	*p* value
**Gender**			
Male	190	0.89 (0.61–1.30)	0.530
Female	394	Ref	
**Age Groups**			
<50 years	216	0.04 (0.02–0.08)	<0.001*
50–74 years	256	0.26 (0.16–0.41)	<0.001*
>74 years	112	Ref	
**ICU stay duration**			
≤3 days	327	0.84 (0.51–1.384)	0.493
4–7 days	169	0.75 (0.43–1.30)	0.298
>7 days	88	Ref	
**Hospital admission to ICU transfer**			
<24 h	380	0.58 (0.404–0.84)	0.003*
≥24 h	204	Ref	
**APACHE-II**			
<15	197	0.00 (NA)	0.994
15–19	132	0.02 (0.01–0.05)	<0.001*
20–28	138	0.24 (0.14–0.4)	<0.001*
≥28	117	Ref	
**SOFA**			
<6	414	0.03 (0.01–0.13)	<0.001*
6–9	157	0.43 (0.09–1.99)	0.278
≥10	13	Ref	
**Number of co-morbidities**			
≤1	303	0.21 (0.14–0.31)	<0.001*
>1	281	Ref	
**ICU**			
Medical ICU	388	3.18 (2.06–4.91)	<0.001*
Surgical ICU	196	Ref	
**Mortality**			
Yes	170	0.12 (0.08–0.18)	<0.001*
No	414	Ref	

OR, odds ratio; CI, confidence interval. Only unadjusted analyses are presented because several variables are components of the mNUTRIC score and adjusted estimates would not be clearly interpretable.

**Table 4.** Unadjusted mortality analysis and adjusted association between mNUTRIC-defined nutritional risk and mortality following ICU admission (*n* = 584).

		Unadjusted	
Variable/category	N	OR (95% CI)	*p* value
**Gender**			
Male	190	0.99 (0.68–1.45)	0.952
Female	394	Ref	
**Age Groups**			
<50 years	216	0.33 (0.19–0.55)	<0.001*
50-74 years	256	0.81 (0.51–1.28)	0.366
>74 years	112	Ref	
**ICU stay duration**			
≤3 days	327	0.60 (0.36–0.98)	0.040*
4-7 days	169	0.72 (0.42–1.24)	0.236
>7 days	88	Ref	
**Hospital admission to ICU transfer**			
0 days	380	0.79 (0.544–1.14)	0.207
≥1 days	204	Ref	
**APACHE-II**			
<15	197	0.02 (0.01–0.04)	<0.001*
15-19	132	0.07 (0.04–0.13)	<0.001*
20-28	138	0.20 (0.12–0.35)	<0.001*
≥28	117	Ref	
**SOFA**			
<6	414	0.09 (0.03–0.31)	<0.001*
6-9	157	0.60 (0.18–2.02)	0.407
≥10	13	Ref	
**Number of co-morbidities**			
≤1	303	0.644(0.45–0.92)	0.016*
>1	281	Ref	
**ICU**			
Medical ICU	388	2.24 (1.48–3.39)	<0.001*
Surgical ICU	196	Ref	
**Nutritional risk by mNUTRIC score**			
Low nutritional risk	408	0.12 (0.08–0.18)	<0.001*
High nutritional risk	176	Ref	

Adjusted OR for mNUTRIC-defined nutritional risk is adjusted for APACHE-II score, number of comorbidities, and ICU type. Adjusted effects for covariates are not shown because the model was designed to estimate the adjusted association between mNUTRIC-defined nutritional risk and mortality.

In unadjusted analysis, variables including older age, longer hospital-to-ICU transfer time, higher APACHE-II score, higher SOFA score, greater comorbidity burden, medical ICU admission and mortality status were associated with high nutritional risk by the mNUTRIC score (Table 3). No adjusted model was reported for nutritional risk because the main associated variables are components of the mNUTRIC score itself, making adjusted estimates difficult to interpret.

In the adjusted mortality model, low nutritional risk based on the mNUTRIC score was associated with lower odds of mortality compared with high nutritional risk (adjusted odds ratio [aOR] 0.51, 95% CI 0.28–0.91). This estimate was adjusted for APACHE-II score, number of comorbidities and ICU type (Table 4).

### Prognostic performance of the mNUTRIC score for ICU mortality

**Figure 2.**
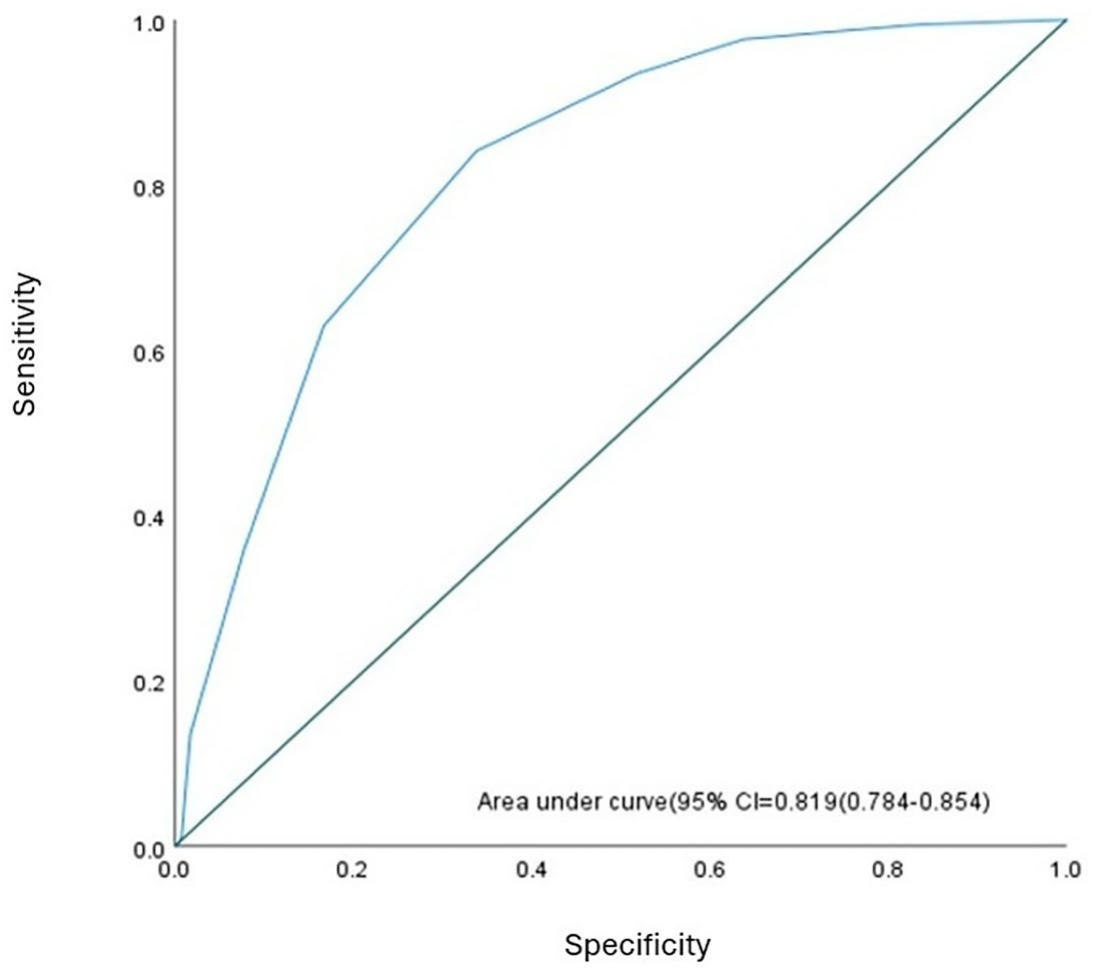
ROC of mNUTRIC score for mortality prediction.

The prognostic ability of the mNUTRIC score to identify patients at increased risk of ICU mortality was evaluated using receiver operating characteristic (ROC) curve analysis. The mNUTRIC score demonstrated good discriminatory power, with an area under the curve (AUC) of 0.819, indicating a strong association between a higher mNUTRIC score and ICU mortality. An optimal cut-off score of > 3.5 (Youden index = 0.50) yielded a sensitivity of 84.1% and a specificity of 66.2% (*p* < 0.001) (Figure 2).

## Discussion

Our study evaluated nutritional risk and mortality outcomes risk in ICU-admitted patients using the mNUTRIC score. The mNUTRIC score serves as a validated tool to identify patients requiring timely and comprehensive nutrition interventions, aiming to enhance their chances of a full recovery [11]. The nutritional risks and applicability of the mNUTRIC score in critically ill patients remain largely unexplored in the context of Pakistan. In our study cohort, the overall mean mNUTRIC score was 3.2, which was lower than the mean scores reported in Indian (4.0) and Jordanian patients [9]. Patients with higher mNUTRIC scores demonstrated high nutritional risk and mortality risk. The overall mortality rate in our study was 29.1%; however, it was notably elevated (62.9%) among patients classified as high risk. A similar trend in mortality was observed among critically ill patients with high mNUTRIC scores admitted to hospitals in Spain and China [16]. Our data suggests that the mNUTRIC score is a reliable and appropriate nutritional risk screening tool for assessing nutritional risk and its association with prognosis in critically ill patients. However, its prognostic association with mortality should be interpreted within the context of illness severity because APACHE-II and SOFA scores are incorporated within the score construct. It is important to acknowledge that the mNUTRIC score does not include a dedicated indicator of patients’ nutritional status per se; rather, it integrates disease severity parameters (APACHE-II and SOFA) alongside age, comorbidity burden and time to ICU transfer. Therefore, the observed association between a high mNUTRIC score and mortality may be substantially driven by underlying disease severity rather than nutritional risk alone. This does not diminish the clinical utility of the score as a prognostic screening tool, but it underscores the importance of interpreting its results as a composite measure of critical illness burden rather than as an independent marker of nutritional status. Future studies incorporating nutritional biomarkers or diagnostic criteria such as the GLIM criteria alongside the mNUTRIC score would help disentangle the independent contribution of nutritional status to the patient outcomes.

Malnutrition has been characterized by slow recovery and unconstrained risk indicator for undesirable clinical outcomes in patients admitted to the ICU [17]. Given that critically ill patients benefit from early and adequate nutrition therapy, the timely identification of those at higher nutritional risk is crucial [18]. Our findings indicate that patients transferred from hospital wards to the ICU within 24 h had a significantly lower nutritional risk compared to those transferred after 24 h. This suggests that early identification of critical illness and timely escalation of care may play a protective role against nutritional deterioration. Delays in ICU transfer may result in prolonged periods of inadequate nutritional support or increased metabolic stress, thereby contributing to a higher nutritional risk. These results underscore the importance of timely clinical decision-making and streamlined transfer protocols to optimize patient outcomes, including nutritional status.

The mNUTRIC score demonstrated strong prognostic performance for mortality, presenting an AUC of 0.81, comparable to studies conducted in Iran (AUC 0.80) [19], Brazil (AUC 0.79) [18], South Korea (AUC 0.75) [20] and the Netherlands (AUC 0.76) [21]. Although our study design was cross-sectional, the observed association between higher mNUTRIC scores and increased ICU mortality aligns with previous studies [10,22]. These findings support the utility of the mNUTRIC scores in identifying patients at a greater risk of mortality in critically ill patients. Implementing nutritional interventions tailored to these scores may contribute to mitigating the nutritional risk and improving clinical outcomes within this population.

Significant correlations between mortality and morbidity prognostic markers (such as SOFA and APACHE II scores) with malnutrition indicators (mNUTRIC scores) provide substantial clinical information. Our results indicated that higher SOFA and APACHE-II scores were significantly associated with both nutritional risk and higher mortality rates. Similar SOFA and APACHE-II score trends have been reported in previous studies assessing ICU mortality [23,24]. Given the heightened risk of ICU mortality among patients with elevated SOFA and APACHE-II scores, the implementation of immediate and targeted nutritional interventions is strongly recommended.

In this study, 30% of patients were identified as having a high nutritional risk, which is lower than the 56% reported in a previous study conducted in Pakistan [7]. This was probably because our study was conducted in a semi-government hospital, where an ICU-trained qualified dietician is employed, who specifically looks after the dietary management of all the patients admitted to the ICU. The presence of multiple comorbidities is an established factor that significantly increases the nutritional risk and mortality in critically ill patients [25–28]. Consistent with this, our study also found a higher risk among patients with concurrent medical conditions such as DM, HTN and ischemic heart disease. Patients with a higher number of comorbidities are more likely to experience progressive health deterioration over time, increasing their likelihood of admission to the medical ICU. This may explain the elevated mNUTRIC scores observed among admitted patients. Therefore, early nutritional risk screening and the implementation of appropriate nutritional support should be standard procedures for critically ill patients in ICUs.

A high nutritional risk was more prevalent among the geriatric population (aged >65 years). Older patients also exhibited an increase in mean APACHE II and SOFA scores, consistent with the findings of a previously published regional study. In contrast, younger patients demonstrated a lower mean mNUTRIC score and a reduced mortality rate, aligning with the 14.7% lower mortality rate observed among individuals under 50 years of age in a study by Kumar et al. [29]. Previous research work has established a significant association between age and the nutritional status of hospitalized patients, as well as the nutritional risk in critically ill patients [30]. The findings of our study further endorse this association.

## Limitations

There are several limitations to our study. First, the mNUTRIC score is a nutritional risk screening tool and does not diagnose malnutrition. We did not apply formal diagnostic criteria such as the Global Leadership Initiative on Malnutrition (GLIM) framework; therefore, our findings should be interpreted as nutritional risk stratification rather than direct malnutrition diagnosis. Second, primary ICU admission diagnoses, such as sepsis, acute respiratory failure, trauma, malignancy, gastrointestinal bleeding and post-surgical admission, were not systematically collected. This is an important limitation because admission diagnosis may influence both nutritional risk and mortality and may represent an unmeasured confounder. Similarly, we did not have data on cognitive disorders such as dementia or Alzheimer’s, which are common in elderly patients and may affect nutritional status and clinical outcomes. This may have influenced our findings, particularly in the older age group. Third, we did not collect detailed information on nutritional interventions, caloric or protein delivery, route of feeding, timing of nutritional support or interruptions in feeding; therefore, we could not evaluate whether nutritional support modified the association between mNUTRIC-defined risk and mortality. Fourth, biochemical and inflammatory markers, including albumin, prealbumin, C-reactive protein and other relevant biomarkers, were not analysed. Fifth, duration of mechanical ventilation and detailed surgical intervention data were not evaluated. Finally, this was a single-centre study, which may limit generalizability to other ICU settings. Future studies should incorporate formal malnutrition diagnostic criteria, primary ICU diagnoses, nutritional intervention data, biochemical markers and multicentre designs to better clarify the independent contribution of nutritional status to ICU outcomes. These limitations restrict the depth of our findings, and future research should address these gaps by including comparative tool analyses, nutritional intervention data, and relevant biochemical parameters to provide a more holistic understanding of nutritional risk and its impact on patient outcomes in ICU settings.

## Conclusion

Our study confirms the applicability of the mNUTRIC score as a reliable nutritional risk screening tool for assessing nutritional risk mortality among ICU patients in a tertiary care hospital in Rawalpindi, Pakistan. While the prognostic value of the mNUTRIC score is well-established internationally, our findings provide important local evidence supporting its use in low-resource settings. Implementing targeted and enhanced nutritional interventions for patients with elevated mNUTRIC scores may contribute to improved survival outcomes. This underscores the need for routine nutritional risk screening and implementation of targeted nutritional interventions to improve the patient outcomes in similar contexts. However, nutritional risk assessment would be more robust with additional comparisons to other validated nutritional screening tools. Future studies should address these gaps to further validate and optimize nutritional risk assessment in these settings.

## Supplementary Material

Supplemental Material

## Data Availability

The dataset used and/or analysed during the study are available from the corresponding author on request.
