# Supplementary material for: Assessing nutritional risk and its association with mortality in ICU patients using the modified NUTRIC score: evidence from a tertiary care hospital
Source: Ann Med. 2026 Jun 18;58(1):2690708. doi: 10.1080/07853890.2026.2690708 (PMC13288537; doi:10.1080/07853890.2026.2690708)
Supplement: Supplemental Material [file IANN_A_2690708_SM7165.docx]

**Table-S1: Association of risk by mNUTRIC score and mortality**

|  | **Risk by mNUTRIC score** | | | **Mortality** | | |
| --- | --- | --- | --- | --- | --- | --- |
|  | **Low Risk** | **High Risk** | **p-value** | **Yes** | **No** | **P-value** |
| **Gender** |  |  |  |  |  |  |
| Male | 136(33.3) | 54(30.7) | 0.530 | 55(32.4) | 135(32.6) | 0.952 |
| Female | 272(66.7) | 122(69.3) |  | 115(67.6) | 279(67.4) |  |
| **Age Groups** |  |  |  |  |  |  |
| <50 years | 200(49) | 16(9.1) | <0.001* | 38(22.4) | 178(43) | <0.001* |
| 50-74 years | 170(41.7) | 86(48.9) |  | 88(51.8) | 168(40.6) |  |
| >74 years | 38(9.3) | 74(42) |  | 44(25.9) | 68(16.4) |  |
| **ICU stay duration** |  |  |  |  |  |  |
| ≤3 days | 228(55.9) | 99(56.3) | 0.580 | 86(50.6) | 241(58.2) | 0.114 |
| 4-7 days | 122(29.9) | 47(26.7) |  | 51(30) | 118(28.5) |  |
| >7 days | 58(14.2) | 30(17) |  | 33(19.4) | 55(13.3) |  |
| **Hospital to ICU duration** |  |  |  |  |  |  |
| 0 days | 281(68.9) | 99(56.3) | 0.003* | 104(61.2) | 276(66.7) | 0.206 |
| ≥1 days | 127(31.1) | 77(43.8) |  | 66(38.8) | 138(33.3) |  |
| **APACHE-II** |  |  |  |  |  |  |
| <15 | 197(48.3) | 0(0) | 0.001* | 10(5.9) | 187(45.2) | <0.001* |
| 15-19 | 121(29.7) | 11(6.3) |  | 22(12.9) | 110(26.6) |  |
| 20-28 | 68(16.7) | 70(39.8) |  | 51(30) | 87(21) |  |
| ≥28 | 22(5.4) | 95(54) |  | 87(51.2) | 30(7.2) |  |
| **SOFA** |  |  |  |  |  |  |
| <6 | 359(88) | 55(31.3) | <0.001* | <0.001* | 343(82.9) | <0.001* |
| 6-9 | 47(11.5) | 110(62.5) |  | 90(52.9) | 67(16.2) |  |
| ≥10 | 2(0.5) | 11(6.3) |  | 9(5.3) | 4(1.0) |  |
| **Co-morbids** |  |  |  |  |  |  |
| Diabetes mellitus | 119(29.2) | 86(48.9) | <0.001* | 69(40.6) | 136(32.9) | 0.075 |
| Hypertension | 139(34.1) | 127(72.2) | <0.001* | 93(54.7) | 173(41.8) | 0.004* |
| Asthma | 17(4.2) | 6(3.4) | 0.666 | 4(2.4) | 19(4.6) | 0.207 |
| Chronic kidney disease | 36(8.8) | 41(23.3) | <0.001* | 30(17.6) | 47(11.4) | 0.041* |
| Ischemic heart disease | 139(34.1) | 127(72.2) | <0.001* | 93(54.7) | 173(41.8) | 0.004* |
| Sepsis | 14(3.4) | 12(6.8) | 0.069 | 12(7.1) | 14(3.4) | 0.050* |
| **No. of Co-morbidities** |  |  |  |  |  |  |
| ≤1 | 257(63) | 46(26.1) | <0.001* | 75(44.1) | 228(55.1) | 0.016* |
| >1 | 151(37) | 130(73.9) |  | 95(55.9) | 186(44.9) |  |
| **ICU** |  |  |  |  |  |  |
| Medical ICU | 243(59.6) | 145(82.4) | <0.001* | 133(78.2) | 225(61.6) | <0.001* |
| Surgical ICU | 165(40.4) | 31(17.6) |  | 37(21.8) | 159(38.4) |  |

Chi-square/fisher exact test was applied.

p-value≤0.05 were considered as significant.

*Significant at 0.05 levels
